# The Archaeal Elongation Factor EF-2 Induces the Release of aIF6 From 50S Ribosomal Subunit

**DOI:** 10.3389/fmicb.2021.631297

**Published:** 2021-03-24

**Authors:** Giada Lo Gullo, Maria Luisa De Santis, Alessandro Paiardini, Serena Rosignoli, Alice Romagnoli, Anna La Teana, Paola Londei, Dario Benelli

**Affiliations:** ^1^Department of Cellular Biotechnologies and Haematology, Sapienza University of Rome, Rome, Italy; ^2^Department of Molecular Medicine, Sapienza University of Rome, Rome, Italy; ^3^Department of Biochemical Sciences, Sapienza University of Rome, Rome, Italy; ^4^Department of Life and Environmental Science, New York-Marche Structural Biology Center (NY-MaSBiC), Polytechnic University of Marche, Ancona, Italy

**Keywords:** IF6, EF2, ribosome, *Sulfolobus solfataricus*, protein synthesis, SBDS

## Abstract

The translation factor IF6 is a protein of about 25 kDa shared by the Archaea and the Eukarya but absent in Bacteria. It acts as a ribosome anti-association factor that binds to the large subunit preventing the joining to the small subunit. It must be released from the large ribosomal subunit to permit its entry to the translation cycle. In Eukarya, this process occurs by the coordinated action of the GTPase Efl1 and the docking protein SBDS. Archaea do not possess a homolog of the former factor while they have a homolog of SBDS. In the past, we have determined the function and ribosomal localization of the archaeal (*Sulfolobus solfataricus*) IF6 homolog (aIF6) highlighting its similarity to the eukaryotic counterpart. Here, we analyzed the mechanism of aIF6 release from the large ribosomal subunit. We found that, similarly to the Eukarya, the detachment of aIF6 from the 50S subunit requires a GTPase activity which involves the archaeal elongation factor 2 (aEF-2). However, the release of aIF6 from the 50S subunits does not require the archaeal homolog of SBDS, being on the contrary inhibited by its presence. Molecular modeling, using published structural data of closely related homologous proteins, elucidated the mechanistic interplay between the aIF6, aSBDS, and aEF2 on the ribosome surface. The results suggest that a conformational rearrangement of aEF2, upon GTP hydrolysis, promotes aIF6 ejection. On the other hand, aSBDS and aEF2 share the same binding site, whose occupation by SBDS prevents aEF2 binding, thereby inhibiting aIF6 release.

## Introduction

The process of protein synthesis is conserved in all living organisms and involves ribosomes, mRNA, and different translation factors. Although the overall size of archaeal ribosomes is similar to that of bacterial ones, their components have a closer homology to those of eukaryotic ribosomes. Indeed, as regards the ribosomal proteins (r-proteins), 33 are common to Archaea and Eukarya (A/E), while of the 34 r-proteins conserved in all three domains, the archaeal and eukaryotic homologs are more similar to each other than to the corresponding bacterial r-proteins (Lecompte et al., 2002; Yutin et al., 2012). Besides, the complexity of archaeal translation is also supported by the larger-than-bacterial number of translation factors, notably translation initiation factors (Dennis, 1997; Benelli and Londei, 2011; Gäbel et al., 2013). The protein known as a/eIF6, a small monomeric polypeptide of about 25 kDa, is one of the translation factors shared by the Archaea and the Eukarya to the exclusion of Bacteria.

In eukaryotes, eIF6 was classified as a translation initiation factor for its ribosome anti-association activity. Indeed, early *in vitro* studies demonstrated the capacity of the protein to bind to the 60S subunit inhibiting its association with the 40S particle (Russell and Spremulli, 1979; Valenzuela et al., 1982). Subsequent structural data showed that eIF6 binds the sarcin-ricin loop (SRL), uL14, and eL24 on the intersubunit face of the large ribosomal subunit preventing ribosomal subunit joining (Gartmann et al., 2010; Klinge et al., 2011; Weis et al., 2015). Genetic studies in *Saccharomyces cerevisiae* showed that eIF6 has a function in the biogenesis and nuclear export of pre-60S subunits (Basu et al., 2001). Later studies confirmed that the removal of eIF6 from the 60S subunit is a late event of ribosome biogenesis and that this step requires the combined action of the GTPase Efl1 and SBDS (Bécam et al., 2001; Menne et al., 2007; Finch et al., 2011; Wong et al., 2011). Specifically, these two factors collaborate to a final quality control assessment for the integrity of the P-site and the GTPase center of the 60S subunit. In mammalian cells, the dislodgement of human eIF6 from the 60S subunit is also described by another model that requires the phosphorylation of the protein on residue S235 by PKCβII kinase recruited on the ribosomes by the receptor for activated C kinase 1 (RACK1) (Ceci et al., 2003). In Archaea, the eIF6 homolog shows a high degree of tertiary structure similarity. Indeed, the A/E factors display a conserved pentein fold (Groft et al., 2000) and this trait suggests that the proteins share a core function conserved in the eukaryal/archaeal line. Indeed, we demonstrated that, similarly to eukaryotes, aIF6 binds to the 30S interacting surface of the large ribosomal subunit, impairing the association between the two subunits (Benelli et al., 2009). Moreover, structural studies confirmed that the ribosome binding site of IF6 is the same as that of its eukaryotic counterpart (Greber et al., 2012).

To date, the molecular mechanism inducing the release of aIF6 from 50S subunits has not yet been determined in Archaea. Phylogenetic analysis of archaeal genomes showed that the ortholog of Efl1 is absent. However, Efl1 is highly homologous to the eukaryotic elongation factor 2 (eEF-2) since it displays the basic organization of a translocation factor composed of a five-domain architecture including the G domain. Moreover, Efl1 can compete with eEF-2 for ribosome binding resulting in the inhibition of the eEF-2 ribosome-dependent GTPase activity (Graindorge et al., 2005). Conversely, SBDS protein is highly conserved in Archaea and Eukaryotes. In humans, mutations of the SBDS gene are associated with the Schwachman–Diamond syndrome (SDS, OMIM 260400), an autosomal recessive disorder. Genetic and biochemical data from different organisms and SDS patient-derived cells support the hypothesis that SBDS is a human ribosomopathy caused by the impaired release and recycling of eIF6 from late cytoplasmic pre-60S ribosomal subunits (Finch et al., 2011; Burwick et al., 2012). In Archaea, the SBDS orthologs are located in a super-operon that encodes proteins constituting the exosome complex and *in vitro* studies have suggested that archaeal SBDS might be involved in RNA metabolism (Koonin et al., 2001; Luz et al., 2010).

In this work, we analyzed the role of both aEF2 and aSBDS in the release of archaeal IF6 from the large ribosomal subunit. Our results suggest that, similarly to eukaryotes, the release of aIF6 from the 50S subunit is a GTPase-dependent mechanism. The involved GTPase is the elongation factor 2 (aEF-2) which is necessary and sufficient to promote aIF6 detachment from the 50S subunit. However, the system does not appear to depend on aSBDS which instead has an inhibitory effect on the detachment of aIF6. To structurally interpret our data, we performed a molecular modeling of the complex aEF2-aSBDS-50S. The results suggest that the binding sites of aEF-2 and aSBDS on 50S subunit overlap. This model would justify the inhibitory effect of aSBDS on aEF2 GTPase activity through a competitive binding mechanism.

## Materials and Methods

### Cloning of the *S. solfataricus* aSBDS and aEF2 Genes and Isolation of the Recombinant Proteins Under Native Conditions

The aEF-2 gene was PCR-amplified from *S.so.* genomic DNA using two synthetic DNA primers constructed on the sequence of the corresponding gene (*SSO0728*). Primer sequences used for aEF2 cloning were as follows: forward primer aEF2-*Nco*I (5′-TTTTTCCATGGCTTGCCTAGATATAAGACAGTAGAGC-3′) and reverse primer aEF2- *Bam*HI (5′- TTTTTGGATCC TCACGACAAGAAATCTTCCACTTTTGG-3′). The amplifica- tion product was then digested with *Nco*I/*Bam*HI enzymes and inserted into the corresponding sites of the pETM11(+) expression plasmid to yield the recombinant pETM-aEF2 (6His) plasmid. The construct adds a tag of six histidine residues to the N-terminus of the recombinant protein. It was sequenced and used to transform *E. coli* strain BL21 (DE3), transformants were grown at 37°C in LB medium containing kanamycin (30 μg/ml). aEF2 expression was induced with 1 mM IPTG at a growth curve of OD_600_ = 0.5 for a further 4 h before harvesting. The cell pellet was resuspended in lysis buffer (50 mM NaH_2_PO_4_, 300 mM NaCl, 10 mM imidazole, pH 8.0) and sonicated. After centrifugation, the cleared lysate underwent a first step of purification for aEF2 by incubating the whole cell lysate at 70°C for 15 min to precipitate mesophilic *E. coli* proteins. Recombinant aEF-2 was purified by affinity chromatography on Ni–NTA agarose (Qiagen) and eluted under native conditions. The elution fraction was precipitated adding (NH_4_)_2_SO_4_ at 70% of saturation, dialyzed against storage buffer (30 mM NH_4_Cl, 20 mM Tris/HCl, pH 8.0) and stored at −80°C in aliquots. The open reading frame of *SSO0737* gene coding aSBDS protein was amplified using forward (5′-TTTTTTTAT GCTAGCATGACGAAGGAGCGTGATTATG-3′) and reverse primer (5′-CATGGTATGCTCGAGTCATCTCACTTGCAATAC TTTAAC-3′) containing *Nhe*I and *Xho*I restriction site, respectively. The amplification product was then digested with *Nhe*I/*Xho*I enzymes and inserted into the corresponding sites of the pRSETB expression plasmid (Novagen) to yield the recombinant pRSETB-aSBDS (6His) plasmid. The construct adds a tag of six histidine residues to the N-terminus of the recombinant protein. It was sequenced and used to transform *E. coli* strain BL21 (DE3). The procedure for its expression and purification was the same described above for aEF2 excepted for the use of ampicillin instead of kanamycin as selector of cells containing the plasmid with the PCR insert. The purified recombinant protein aSBDS was dialyzed against the storage buffer containing 20 mM TEA pH 7.4, 10 mM KCl, 5% glycerol, and preserved in aliquots at −80°C.

### Preparation of *S. solfataricus* Cellular Extracts and Cellular Fractions

Whole cell extracts were prepared starting from frozen *Sulfolobus solfataricus* cell pellets following the procedure previously described (Benelli and Londei, 2007). Crude cellular lysates (S30) were centrifuged in a Beckman Ti 50 rotor at 100,000×*g* and 4°C for 3 h to separate ribosomes from a supernatant (S-100) containing total cellular tRNAs and ribosome free cytoplasmatic proteins. The pellet of ribosomes (termed “crude” ribosomes, CRs) was resuspended in the extraction buffer (20 mM Tris/HCl pH 7.4, 10 mM Mg(OAc)_2_, 40 mM NH_4_Cl, 1 mM DTT). The proteins of S-100 cell fraction were concentrated, adding ammonium sulfate to 70% saturation. The precipitate was collected by centrifuging 10 min at 15,000 rpm; the pellet was dissolved in the resuspending buffer (20 mM Tris/HCl pH 7.4, 2 mM Mg(OAc)_2_, and 2 mM DTT) and dialyzed against the same buffer. Ribosomes devoid of extrinsic proteins and some translation factors were obtained, resuspending crude ribosome pellet in salt-buffer (20 mM Tris/HCl pH 7.4, 500 mM NH_4_Cl, 10 mM Mg(OAc)_2_, 1 mM DTT), and then loaded on 18% (w/v) sucrose cushion in the same buffer. Then, they were centrifuged in a Beckman Ti 50 rotor at 100,000×*g* for 4 h at 4°C. The final ribosome pellet (termed “high-salt washed” ribosomes, 70S HSW) was resuspended in the extraction buffer containing 3% glycerol. The concentration of the ribosomes was determined by measuring the A_260_ and considering 1 OD_260_ 70S = 40 pmol. The supernatant recovered after the sedimentation of HSW was supplemented with ammonium sulfate at a final concentration of 70% and stirred on ice for about 1 h. The precipitate was collected by centrifuging for 10 min at 15,000 rpm; the pellet was dissolved in the resuspending buffer (20 mM Tris/HCl pH 7.4, 2 mM DTT, 5% glycerol) and dialyzed against the same buffer. This preparation was the high salt wash (HSW).

### Isolation of Ribosomal Subunits

Aliquots of the salt-wash ribosomes (40 A_260_ units) were layered onto preparative 38 ml linear 10−30% (w/v) sucrose density gradients made in the ribosome-suspending buffer (20 mM Tris/HCl, pH 7.0, 40 mM NH_4_Cl, 10 mM Mg(CH_3_COO)_2_, 2.0 mM dithiothreitol). The gradients were centrifuged in a Beckman SW 27 rotor operated at 18,000 rev/min and 4°C for 18 h. Fractions corresponding to the 30S and 50S peaks of A_260_ were separately pooled and the particles therein were precipitated by the addition of two volumes of ethanol. After low-speed centrifugation, the subunit pellets were resuspended in the ribosome extraction buffer containing 10% (v/v) glycerol and stored at −20°C.

### GTP Hydrolysis Assay

The amount of inorganic phosphate released after GTP hydrolysis was monitored by the use of ammonium molybdate in sulfuric acid solution. In these experimental conditions, phosphate reacts with ammonium molybdate to form a yellow phosphorous molybdate complex showing an absorption peak at 660 nm. Measurement of aEF2 GTPase activity was carried out at 65°C for 20 min in a final volume of 0.05 ml containing 20 mM Tris/HCl, pH 7.4, 10 mM KCl, and 10 mM MgCl_2_. The amount of protein used in each reaction is described in the legend of the corresponding figure. After terminating the reaction, the volume was brought up to 0.3 ml with the reaction buffer. This was followed by the addition of 0.7 ml of a reagent containing one part of 10% ascorbic acid and six parts of 0.42% ammonium molybdate ⋅4H_2_O (prepared in 1 N H_2_SO_4_). After thoroughly mixing, the content was incubated at 45°C for 20 min permitting the color development that was read at 660 nm.

### *In vitro* Translation

*In vitro* translation was performed by programming a whole cell lysate prepared as described before (Benelli and Londei, 2007). The samples contained in a final volume of 100 μl: 10 mM KCl, 20 mM TEA/HCl (pH 7.4), 20 mM MgCl_2_, 3 mM ATP, 1 mM GTP, 4 μg of *S. solfataricus* total tRNA, 0.55 mg of S30 extract, and 4 μg of *in vitro* transcribed 104 mRNA. The samples were incubated for 45 min at 70°C. At the end of the reaction, fixation on ice with 1% formaldehyde for 30 min was performed to stabilize 70S ribosomes which are easily dissociated in *S. solfataricus* and the samples were layered on linear, 10–30% sucrose gradients containing 10 mM KCl, 20 mM TEA/HCl pH 7.4, and 20 mM MgCl_2_. The gradients were centrifuged at 36,000 rpm for 4 h and 30 min in a Beckman SW41 rotor at 4°C and 36,000 rpm for 4 h and unloaded while monitoring absorbance at 260 nm.

### Sucrose Gradient Analysis

The association of recombinant and/or endogenous proteins to ribosomal subunits was investigated by fractionating different samples on sucrose density gradient and then probing each fraction for the presence of the proteins by western blot with specific antibodies. Specifically, at the end of each reaction, the samples were layered on linear 10–30% sucrose gradients containing 10 mM KCl, 20 mM TEA-HCl (pH 7.5), and 20 mM MgCl_2_; these were centrifuged in a Beckman SW41 rotor at 4°C and 36,000 rpm for 4 h or at 18,000 rpm for 17–18 h. After centrifugation, the gradients were unloaded while monitoring absorbance at 254 nm with the EM-1 Econo UV absorbance instrument (Bio-Rad). The individual fractions (0.5 ml) were collected in single tubes and precipitated adding 1/100 volume of 2% Na-deoxycholate and 1/10 of trichloroacetic acid 100%, vortexed, and let sit over-night at 4°C. Then, the samples were centrifuged 15′ at 13,000×*g*, the protein pellets were resuspended in 20–40 μl of 1X Laemmli Sample Buffer, separated by 15% SDS–PAGE, and electroblotted to nitrocellulose membrane. On the basis of the protein analyzed, we probed the membrane with house made rabbit polyclonal antibodies (antibody against aSBDS and aIF6) or a 6x-His Tag monoclonal antibody (Thermo Fisher Scientific).

### Western Blot Analysis

The protein concentration of different cell fractions was quantified using the Bradford assay. Equal amounts of protein samples were subjected to SDS-PAGE and transferred to nitrocellulose membrane (Amersham Protran-GE Healthcare, Little Chalfont, Buckinghamshire, United Kingdom). After blocking non-specific binding of antibody with 5% non-fat milk, blots were probed with one of the following antibodies: anti-aIF6 polyclonal rabbit antibodies (1:5,000), anti-aSBDS polyclonal rabbit antibodies (1:10,000), 6×-His Tag Monoclonal Antibody (4E3D10H2/E3; Thermo Fisher Scientific). Primary antibodies were detected by binding horseradish peroxidase (HRP)-conjugated goat anti-rabbit IgG-HRP (sc-2004; Santa Cruz Biotechnology), goat anti-mouse IgG-HRP (sc-2005; Santa Cruz Biotechnology), and using an enhanced chemiluminescent visualization system (ECL Western Blotting Substrate, Thermo Fisher Scientific-Pierce Biotechnology, Rockford, IL, United States). 6×-His Tag Monoclonal Antibody and secondary antibodies were diluted according to the manufacturer instructions. The images were captured by a BioRad ChemiDoc. MP Imaging system (Bio-Rad, Hercules, California, United States).

### Protein Structure Analysis, Modeling, and Docking

The Combinatorial Extension (Shindyalov and Bourne, 1998) and PyMOL (Schrodinger, 2013) tools were used for structure superposition and visualization, respectively. Modeler v.9.9 (Sali and Blundell, 1993) and its graphical interface PyMod (Bramucci et al., 2012; Janson et al., 2017; Janson and Paiardini, 2020) were used for homology modeling purposes; models were validated using standard tools (Laskowski et al., 1996; Wiederstein and Sippl, 2007). The Phyre v2.0 server was used for finding candidate templates for homology modeling (Bennett-Lovsey et al., 2008). Prediction of the potential presence of protein-protein interaction sites was carried out with the consensus method implemented in meta-PPISP at the web site http://pipe.scs.fsu.edu/meta-ppisp (Qin and Zhou, 2007). Protein–protein docking was carried out starting from the original position of the homologous protein complexes and refined using the ClusPro method available at the server http://cluspro.bu.edu (Kozakov et al., 2010).

### Size-Exclusion Chromatography (SEC)

Allyl dextran-based size-exclusion gel (Sephacryl S-300, GE Healthcare) was used as stationary phase. The gel column was prepared by filling a 15 cm long column with an appropriate amount of allyl dextran-based size-exclusion gel dilute 1:1 with the following solution buffer: 10 mM KCl, 20 mM TEA-HCl (pH 7.5), and 20 mM MgCl_2_. The flow rate of the running buffer was 1 ml/min and the presence of molecules along the flow was monitored by reading the absorbance at 254 nm with the EM-1 Econo UV absorbance instrument (Bio-Rad). The speed of the recording pare was set to 1 cm/min.

### Statistical Analysis

All data shown represent at least three independent experiments. Western blot bands intensities were captured and analyzed by a ChemiDoc MP Imaging system (Bio-Rad, Hercules, California, United States). Values represent the mean ± SEM. *P*-values listed represent a two-tailed Student’s *t*-test *P*-value. *P* < 0.05 was considered statistically significant.

## Results

### aIF6 Is Released From the 50S Subunits Through a Ribosome-Dependent GTPase Activity

The well-known role of the a/eIF6 protein as a ribosome anti-association factor leads to the assumption that the factor has to be released from the large ribosomal subunits to permit their access to the elongation cycle. Indeed, in our previous work we showed that lysates programmed for protein synthesis triggered the dissociation of aIF6 from the 50S subunits (Benelli et al., 2009). To elucidate the mechanism inducing aIF6 release we focused our attention on a simplified system consisting of just whole ribosomes. Specifically, we used one of the following fractions: (1) crude 70S, i.e., ribosomes obtained by high-speed centrifugation of whole cell lysates; (2) high salt purified ribosomes (70S HSW), i.e., purified ribosomes washed with a high salt buffer and devoid of most translation factors; (3) purified 50S subunits.

**FIGURE 1:**
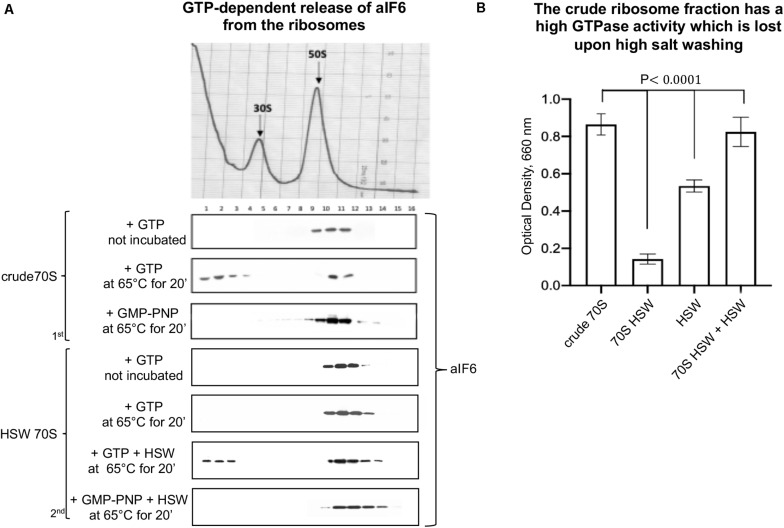
The GTPase activity of extra-ribosomal 70S fraction induces the release of aIF6 from the 50S subunits. **(A)** Density gradient fractionation of crude 70S or HSW 70S in a mixture containing GTP or GMP-PNP and incubated at 65°C for 20 min. The curly brackets group experiments made with the same ribosome preparation. The distribution of aIF6, shown at the bottom of each gradient profile, was revealed by western blotting of the individual fractions with the anti-aIF6 antibodies. The distribution of ribosomal subunits was identified by the optical scans at OD254 nm of the gradients. **(B)** GTPase activity of different cell fractions was determined reading at 660 nm the amount of the phosphate/molybdate complex formed after GTP hydrolysis as described in the “Materials and Methods” section. Data are presented as mean value ± *SD* (*n* = 4). A representative image of at least three independent sucrose density experiments is shown for each analysis.

Initially, we performed *in vitro* studies incubating crude ribosomes in presence of GTP at 65°C for 15 min. We observed that under these conditions a substantial fraction of bound aIF6 was released (Figure 1A, 1st panel). This showed that ongoing translation is not required for aIF6 detachment. However, when the experiment was repeated using HSW 70S instead of crude ribosomes, aIF6 was not released, suggesting that the high-salt washing of ribosomes removed some factor essential for aIF6 detachment. Indeed, when the proteins removed by washing (HSW) were added back to the reaction mix, aIF6 release was again observed (Figure 1A, 2nd panel). Significantly, in all of the previous experiments, substituting GTP with GMP-PNP (a non-hydrolyzable analog of GTP) blocked aIF6 release, demonstrating that it was dependent on the hydrolysis of GTP. Hence, these preliminary results suggested that, similarly to the eukaryotes, some GTPase was implicated in removing aIF6 from the 50S subunits. Indeed, the GTPase assays shown in Figure 1B indicate that the crude ribosome fraction has a high GTPase activity which is lost upon high salt washing. Addition of HSW proteins to the washed 70S restored their GTPase activity to levels comparable to those of crude 70S. Overall, these experiments further support the idea that the detachment of aIF6 from 50S subunits requires the action of some critical GTPases loosely associated with the crude 70S ribosomes.

### Ribosome-Dependent GTPase Activity of aEF-2 Induces the Release of aIF6

As said before, archaea do not possess homologs of the specialized GTPase Efl1. However, Efl1 is a close homolog of elongation factor 2 (EF-2), which raised the possibility that, in archaea, EF-2 itself could be the GTPase protein implicated in aIF6 detachment.

**FIGURE 2:**
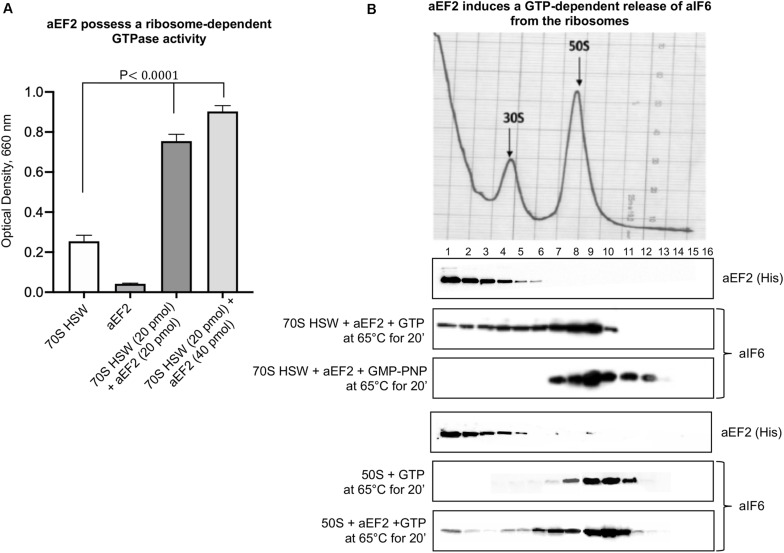
aEF-2-induced release of aIF6 from ribosomes. **(A)** The GTPase activity of recombinant aEF-2 protein was analyzed by incubating 20 or 40 pmol of the protein with 20 pmol of 70S HSW and 1 mM GTP at 65°C for 20 min. At the end of the reaction, the inorganic phosphate released after GTP hydrolysis was revealed as described in the “Materials and Methods” section. Data are presented as mean value ± *SD* (*n* = 4). **(B)** Density gradient fractionation of 70S HSW (70 pmol) or 50S (50 pmol) incubated at 65°C for 20 min in the presence of aEF-2 (70 or 50 pmol) and 1 mM GTP or GMP-PNP. The distribution of aIF6 and recombinant aEF2 was revealed by western blotting of the individual fractions with anti-aIF6 and 6(His) antibodies, respectively. The distribution of the ribosomal subunits was identified by the optical scans at OD254 nm of the gradients. A representative image of at least three independent sucrose density experiments is shown for each analysis.

To verify this surmise, we decided to clone the *Sulfolobus solfataricus* gene *SSO0728* encoding the aEF-2 protein into an expression vector (pETM11+) adding a 6(His)-tag to the N-terminus of the recombinant protein (Supplementary Figure S1A). Upon expression in *E. coli*, the construct produced a recombinant aEF-2 protein devoid of the unique post translational modification specific of eukaryotic and most archaeal translational elongation factor 2 and known as diphthamide (Schaffrath et al., 2014; Narrowe et al., 2018). Therefore, we preliminarily verified whether our recombinant construct possessed a ribosome-dependent GTPase activity. The experiments in Figure 2A show that this was indeed the case, in accordance with previous evidence (de Vendittis et al., 1997). Successively, we analyzed the involvement of aEF-2 in aIF6 detachment from the 50S subunit incubating the HSW 70S in the presence of the recombinant protein at 65°C for 20 min. As shown in Figure 2B, under these conditions, aEF-2 was able to promote the release of aIF6; this ability was dependent on the hydrolysis of GTP, since the presence of GMP-PNP inhibited the reaction. These results were also reproduced using size-exclusion chromatography instead of density-gradient centrifugation (Supplementary Figure S2). Finally, to determine whether the presence of the 30S subunit was required for the aEF-2-induced aIF6 release, we performed the same experiments also using gradient-purified 50S subunits. As shown in the last lane of Figure 2B, aEF-2 was able to induce the release of aIF6 also in this case, suggesting that aIF6 detachment takes place on individual 50S ribosomal subunits that have not yet entered the translation cycle.

### Localization of Archaeal SBDS in *S. solfataricus* Cell Extracts

**FIGURE 3:**
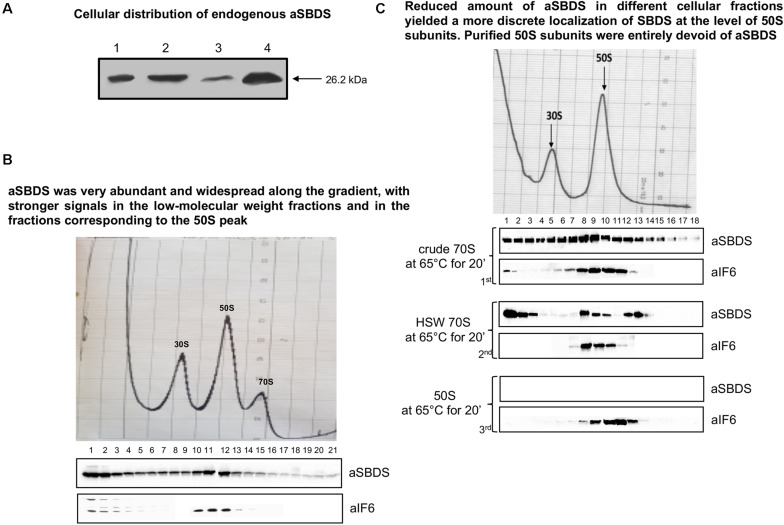
Localization of endogenous aSBDS in a cell lysate. **(A)** Identification of endogenous aSBDS in different cell fractions by western blot: (1) cell extract; (2) crude ribosomes; (3) high-salt washed ribosomes; and (4) post-ribosomal supernatant (HSW). **(B)** Density gradient fractionation, after fixation with HCHO, of cell lysates programmed for translation and incubated at 70°C for 45 min. **(C)** Density-gradient fractionation of: crude 70S (70 pmol) in the 1st panel, HSW 70S (70 pmol) in the 2nd panel, and 50S subunits (50 pmol) in the 3rd panel. Each sample was incubated at 65°C for 20 min in the presence of 1 mM GTP. Braces group experiments made with the same ribosome preparation. The distribution of endogenous aIF6 and aSBDS shown at the bottom of each gradient profile was revealed by western blotting of the individual fractions with the anti-aIF6 and anti-aSBDS antibodies, respectively. In **(B,C)** the distribution of ribosomal subunits was identified by the optical scans at OD254 nm of the gradients. A representative image of at least three independent sucrose density experiments is shown for each analysis.

The experiments described above establish the importance of aEF-2 in removing aIF6 from the 50S ribosomal subunit, thereby enabling the particles to enter the elongation cycle. However, they do not elucidate whether the aSBDS protein retains a conserved evolutionary function, namely if it cooperates with aEF-2 in promoting the release of aIF6 from the 50S subunit. To investigate this point, we cloned the *S. solfataricus* gene *SSO0737* by PCR amplification on genomic DNA, inserted the amplified fragment in the expression plasmid pRSETB, expressed the plasmid in *E. coli* BL21 (DE3), and purified the recombinant protein from cell extracts by differential thermal denaturation and affinity chromatography. This procedure yielded a recombinant aSBDS protein (aSBDS_*r*_) containing a 6xHis tag to its N-terminus that migrated as a single sharp band free of detectable contaminants (Supplementary Figure S3A). The purified protein was used to produce polyclonal antibodies to monitor the cellular distribution of the endogenous protein. When tested on both whole cell lysates and ribosome preparations, the aSBDS antiserum recognized a single polypeptide, which was abundant in the crude 70S but reduced in the HSW ribosomes (Figure 3A).

### Translational Behavior of aSBDS

To investigate the behavior and localization of aSBDS during translation, sucrose density gradient analysis was performed on lysates programmed for protein synthesis as described earlier (Benelli and Londei, 2007). The programmed lysates were incubated at 70°C for 45 min to activate translation and were then fixed with formaldehyde to stabilize 70S ribosomes which are easily dissociated in *S. solfataricus*. As shown in Figure 3B, aSBDS was very abundant and widespread along the gradient, with stronger signals in the low-molecular weight fractions and in the fractions corresponding to the 50S peak. Some signal was also present in high-molecular weight fractions, similar to what was observed in yeast by other authors (Menne et al., 2007). A similar pattern was obtained upon gradient fractionation of crude 70S ribosomes (Figure 3C, 1st panel), while HSW 70S, which contain reduced amounts of aSBDS, yielded a more discrete localization of SBDS at the level of 50S subunits and higher fractions (Figure 3C, 2nd panel). In particular, the peak of SBDS observed in post-50S fractions may be due to the presence of the protein in high-mol-wt complexes formed with some other component present in the ribosome preparations. Artifacts due to precipitation and aggregation of SBDS were ruled out since the same reaction mixture devoid of ribosomes produced a signal of the recombinant aSBDS protein just in the first fractions (Supplementary Figure S3B). Gradient-purified 50S subunits were entirely devoid of aSBDS (Figure 3C, 3rd panel), demonstrating that the protein is not strongly associated with the ribosomes.

### aSBDS Inhibits the GTPase Activity of aEF-2 and the Release of aIF6 From the Ribosomes

**FIGURE 4:**
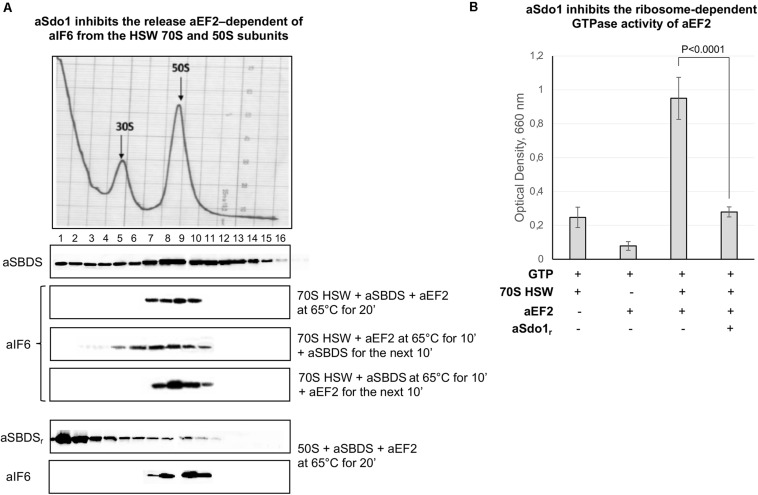
aSBDS inhibits the release of aIF6 induced by aEF2. **(A)** Density gradient fractionation of HSW ribosomes (70 pmol) or 50S subunits (50 pmol) incubated with aSBDS (140 and 100 pmol, respectively), and aEF-2 (140 and 100 pmol, respectively), at 65°C for 20 min. Each sample was incubated in presence of 1 mM GTP. The distribution of aIF6 was revealed by western blotting of the individual fractions with the anti-aIF6 antibodies. The distribution of endogenous and recombinant aSBDS was revealed by western blotting of the individual fractions with the anti-aSBDS antibodies. The distribution of ribosomal subunits was identified by the optical scans at OD254 nm of the gradients. A representative image of at least three independent sucrose density experiments is shown for each analysis. **(B)** GTPase activity of aEF-2 (40 pmol) was analyzed by incubating the recombinant protein in presence/absence of 70S HSW (20 pmol), aSBDS (40 pmol), and 1 mM GTP at 65°C for 20 min. At the end of the reaction, the inorganic phosphate released after GTP hydrolysis was detected, as described in the “Materials and Methods” section. Data are presented as mean value ± *SD* (*n* = 4).

The role, if any, of aSBDS in the release of aIF6 from the large ribosomal subunit was directly investigated by adding the purified protein to a reaction mixture containing 70S HSW and aEF-2. Surprisingly, the presence of aSBDS effectively inhibited the aIF6 release from the ribosomes (Figure 4A, 1st lane). Similar results were also obtained when purified 50S subunits were used (Figure 4A, 4th lane). To get a better insight into this result, we repeated the experiments by adding aSBDS and aEF-2 at different times to the reaction mixture containing 70S HSW. As shown in Figure 4A, addition of SBDS 10 min after the start of the reaction with aEF-2 allowed a limited release of aIF6, while when SBDS was added at the outset and aEF-2 10 min later, aIF6 detachment was completely blocked. Furthermore, GTPase assays showed that aSBDS substantially inhibited the ribosome-dependent GTPase activity of aEF2 (Figure 4B). Upon the whole, the results suggested that aEF-2 and SBDS competed for a same ribosome-binding site, and that only ribosomes devoid of aSBDS were competent for aEF-2-induced aIF6 release.

## Discussion

In this work, the mechanism of release of the translation factor aIF6 from the large ribosomal subunit has been experimentally studied for the first time. Although a final mechanism has not been defined and will require further work, the results obtained have unveiled interesting homologies and differences with the corresponding eukaryotic process. Firstly, we could conclude that aIF6 release from archaeal large ribosomal subunit, similar to eukaryotes, is a GTPase-dependent event. The involved GTPase is the elongation factor 2 (aEF-2) which by itself is necessary and sufficient to induce aIF6 detachment from the ribosomes, even in the absence of ongoing translation. Indeed, we observed the release of aIF6 from the 50S subunits in a reaction mixture containing just high-salt washed 70S, aEF-2, and GTP, without the other components necessary for translation such as tRNAs, mRNA, and translation factors. Since Archaea do not possess a homolog of the GTPase Efl1 involved in the eIF6 release in eukaryotes, a role of aEF-2 in the process had already been suggested both on the basis of the fact that Efl1 is a close homolog of aEF2, and because in eukaryotes Efl1 inhibits the GTPase activity of EF-2, probably because they compete for the same ribosome-binding site (Graindorge et al., 2005; Tanzawa et al., 2018). Indeed, we found that the reaction relied on the GTPase activity of the factor since the presence of GMP-PNP instead of GTP in the reaction inhibited the detachment of aIF6 from the ribosomes. However, release of aIF6 in Archaea does not appear to require the eukaryotic SBDS homolog. Instead, aSBDS seems to have an inhibitory effect on aIF6 detachment, probably because its ribosomal binding site overlaps with that of aEF-2 and the two factors compete for binding.

**FIGURE 5:**
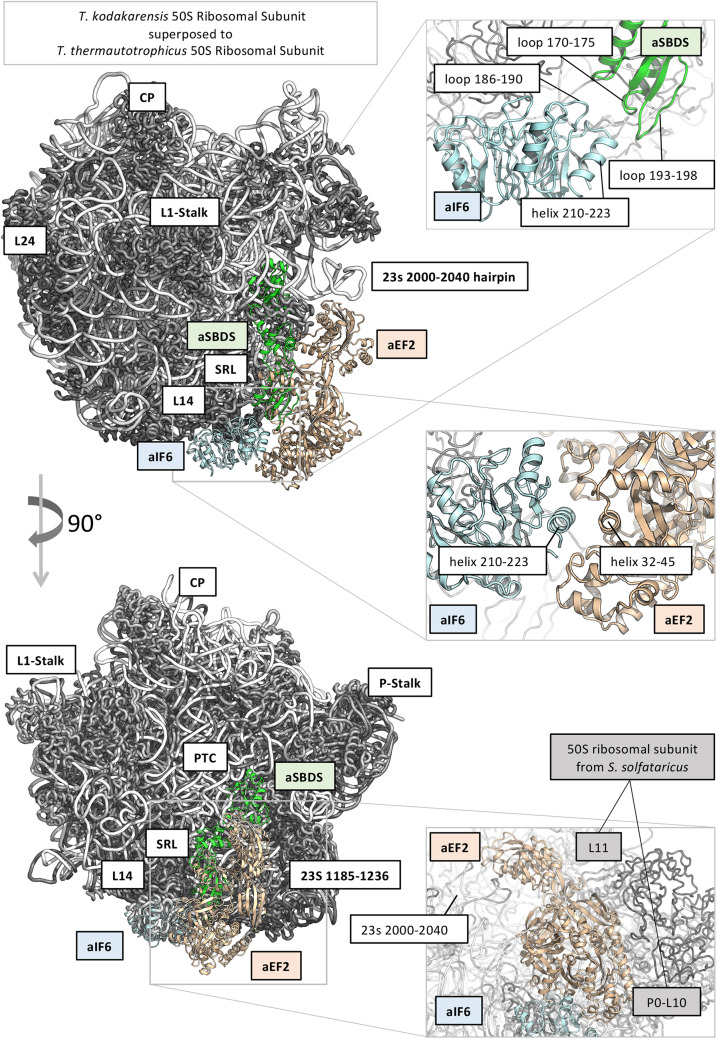
Structural models of translation initiation factor 6 (aIF6), elongation factor 2 (aEF2), and ribosome maturation protein SBDS-like (aSBDS) from *Sulfolobus solfataricus* (cyan, gold, and green cartoons, respectively). The 50S ribosomal subunits from *T. kodakarensis* (PDB 6SKG) (Sas-Chen et al., 2020) and *T. thermautotrophicus* (PDB 4ADX) are shown as the reference in gray (protein) and white (rRNA) ribbons. The approximate position of aIF6 and aEF2 related to the 60S ribosomal subunit is based on the homologous structures from *Dictyostelium discoideum* (PDB 5ANB). The relative position of aEF2 and aSBDS suggests that the two proteins partially overlap and compete for the same binding site on the 50S subunit.

In order to get a structural rationale of the results, we decided to model aIF6 (Uniprot ID: Q980G0) from *S. solfataricus*, based on the very high sequence identity with the homologous structure from *Methanocaldococcus jannaschii* (PDB: 1G61; Sequence identity: 47%), and to model also aSBDS (Uniprot ID: D0KTE1), based on the homologous from *Archaeoglobus fulgidus* (PDB: 1P9Q, sequence identity: 44%) (Savchenko et al., 2005; Figure 5). Moreover, the positions of aIF6 and aSBDS relative to the ribosomal subunit were obtained by superposing the predicted models with the homologous structures of the 60S ribosomal subunit from *Dictyostelium discoideum* (PDB 5ANB), and the 50S ribosomal subunits of *T. kodakarensis* (PDB 6SKG) (Sas-Chen et al., 2020) and *T. thermautotrophicus* (PDB 4ADX). Two loops of aSBDS (residues 170–175; 193–198) are mainly contacting in the model two regions of aIF6 (186–190; 206–210) suggesting that aSBDS could stabilize aIF6 in its interaction with the ribosome (Figure 5, upper right panel). On the other hand, modeling of aEF-2 (Uniprot ID: P30925) using as structural template the crystal structure of the homologous protein from *Pyrococcus horikoshii* (PDB: 5H7J, sequence identity: 50%) (Tanzawa et al., 2018), and its relative position on the ribosomal subunits as previously described, evidenced that aEF-2 is substantially smaller than eEF-2, and lacks an important domain region of eEF2 (PDB: 5ANB), namely 541–821, which is involved in binding and stabilizing SBDS in the eukaryotic complex. aEF-2 is instead stabilized by interactions with the archaeal proteins L10 and L11 (Figure 5, lower right panel), and the archaeal 23s 2,000–2,040 hairpin. A partial overlap and competition are observed between aEF-2 and aSBDS in binding to the ribosome (Figure 5). Our modeling suggests that a tight interaction takes place between aIF6 and aEF-2, as previously observed (Figure 5, central panel). Therefore, it is conceivable that this interaction could be retained also after the conformational transition of aEF-2, upon GTP binding and hydrolysis. The overall effect of such conformational transition of aEF-2 would therefore be the displacement of aIF6 from its bound position on the ribosome.

To verify this, aEF-2 was modeled in its open conformation (based on the crystal structure of *Methanoperedens nitroreducens* EF2, PDB code: 6U45) (Fenwick and Ealick, 2020) and morphed between its open/closed states. Indeed, the model predicts that upon conformational transition of aEF2, aIF6 is displaced from its previous position.

**FIGURE 6:**
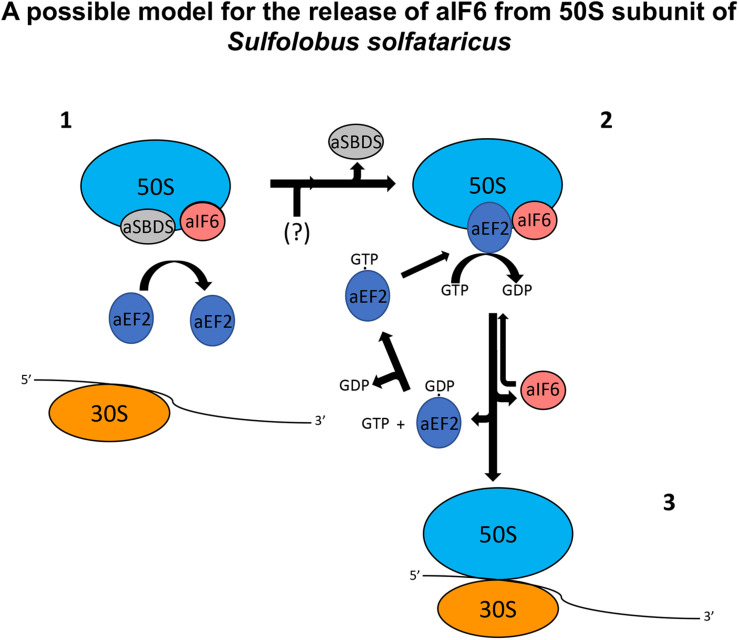
A plausible model depicting the mode of action of aEF-2 on the 50S subunits for the release of aIF6 in *Sulfolobus solfataricus*. The picture represents 50S subunits with the aIF6 and aSBDS proteins bound on it. In the first step, the presence of aSBDS on the ribosomes does not permit the binding of aEF-2 (1). The activity of an unknown factor (here represented with the symbol “?”) induces the release of aSBDS from the ribosomes facilitating the binding of aEF-2 to the 50S subunits (2). The hydrolysis of GTP bound to aEF-2 induces a conformational change in the ribosome and/or in the structure of aEF-2 itself with the consequent release of aIF6. aEF2 bound to GDP dissociates from the ribosomes and the next exchange of GDP/GTP on aEF-2 allows the recruitment of the protein to a new cycle of aIF6 release. Similarly, free aIF6 is ready to bind newly to the large ribosomal subunits while 50S subunits, free of aIF6, can instead complete the translation initiation phase (3).

SBDS has also been shown to share in part the same binding site with the GTPase Efl1 in eukaryotes: however, in the eukaryotic system, the arrival of Efl1 causes a conformational change of SBDS that is in turn required for the ejection of eIF6. In this view, eukaryotic SBDS functions as a cofactor for elongation factor-like GTPase 1 (Efl1). This does not seem to be the case in Archaea where, probably, the aEF-2-dependent detachment of aIF6 has to be preceded by the release of aSBDS from the ribosomes. In Figure 6, we present a model based on the previous results, which proposes a plausible explanation of the interplay among the translation factors in question.

In synthesis, aSBDS and aEF-2 would be two proteins that orchestrate, and participate in a distinct temporal manner to, the formation of a functional 50S. Specifically, aSBDS could be a protein belonging to the class of trans-acting factors known as “placeholders” which temporarily bind selected ribosomal sites until these have achieved a structure appropriate for exchanging the placeholder with another site-specific binding factor (Fenwick and Ealick, 2020). In the present case, the other factor would be aEF-2, whose action as a remover of aIF6 would be hindered by aSBDS until the biogenesis of the particle is completed.

However, the role of archaeal SBDS in the context of ribosome biogenesis or of translation is far from being clear and will require further experimentation to be fully elucidated. A certain amount of evidence would lead to speculate that aSBDS could be a part of the exosome system involved in the maturation of rRNA during ribosome biogenesis. First, in archaea, the aSBDS gene is located in a super-operon that encodes proteins constituting the exosome complex (Supplementary Figure S4). Second, *in vitro* studies have suggested that archaeal SBDS might be involved in RNA metabolism affecting RNA-exosome activity (Luz et al., 2010). Third, our present results show that aSBDS is very abundant in *Sulfolobus* cells and that it is widely distributed on density-gradient fractions, apparently being also included in high-mol-wt complexes of unknown composition. Indeed, there are data in literature showing that some of *Sulfolobus solfataricus* exosome components, in particular Rrp41, show a sedimentation pattern not unlike what we observed for aSBDS (Warren, 2018).

Upon the whole, the previous considerations could lead to conceive a tentative scenario, where the ancestral function of SBDS (retained in present-day Archaea) was to participate in the maturation of pre-rRNA on the large ribosomal subunit. SBDS would remain on the ribosome until this task was completed, also obstructing the binding site for ribosome-dependent GTPases such as aEF-2, thus preventing the premature release of the anti-association factor aIF6. In a later evolutionary stage, in the eukaryotic lineage, both SBDS and the RNA-exosome complex retained a pivotal role for the maturation of the pre-ribosomes but in two distinct temporal steps. A specific GTPase dedicated to eIF6 detachment also emerged (Menne et al., 2007; Witharana et al., 2012; Espinar-Marchena et al., 2017). The mechanism triggering the detachment of aSBDS from the archaeal ribosome remains to be understood; conceivably, it could be a conformational change induced by an unknown GTPase that accompanies the final maturation of the large subunit. Future research will hopefully shed light on this very interesting process.

## Data Availability Statement

The original contributions presented in the study are included in the article/Supplementary Material, further inquiries can be directed to the corresponding author/s.

## Author Contributions

DB, PL, and AL conceived and designed the experiments. DB, GL, MD, and AR performed the experiments. DB, PL, AL, and AP analyzed the data. DB, PL, and AP wrote the manuscript. All authors contributed to the article and approved the submitted version.

## Conflict of Interest

The authors declare that the research was conducted in the absence of any commercial or financial relationships that could be construed as a potential conflict of interest.
