# Supplementary figures and images for: The Archaeal Elongation Factor EF-2 Induces the Release of aIF6 From 50S Ribosomal Subunit
Source: Front Microbiol. 2021 Mar 24;12:631297. doi: 10.3389/fmicb.2021.631297 (PMC8024482; doi:10.3389/fmicb.2021.631297)

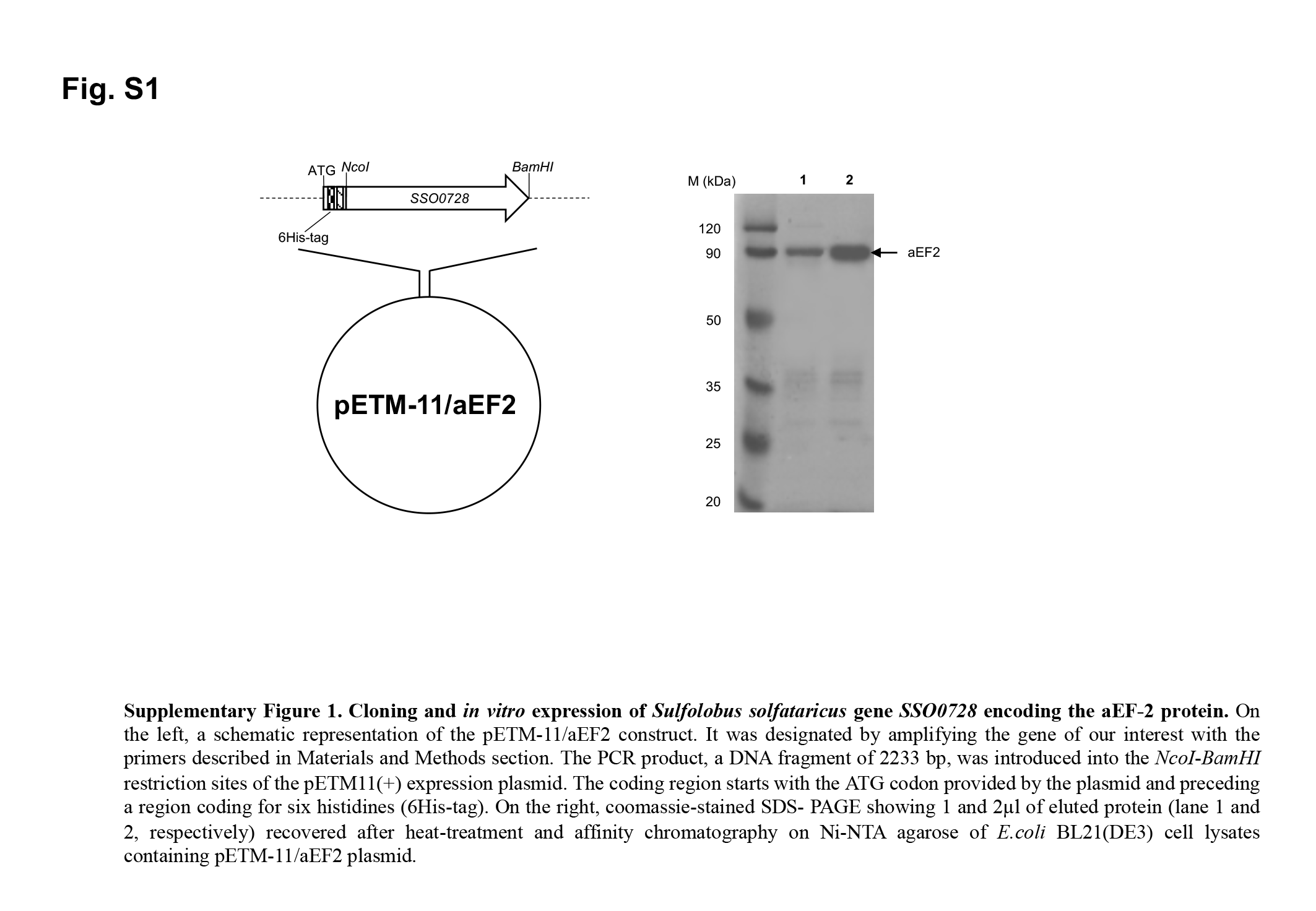

Supplement: Supplementary file 1 [file Image_1.TIF]

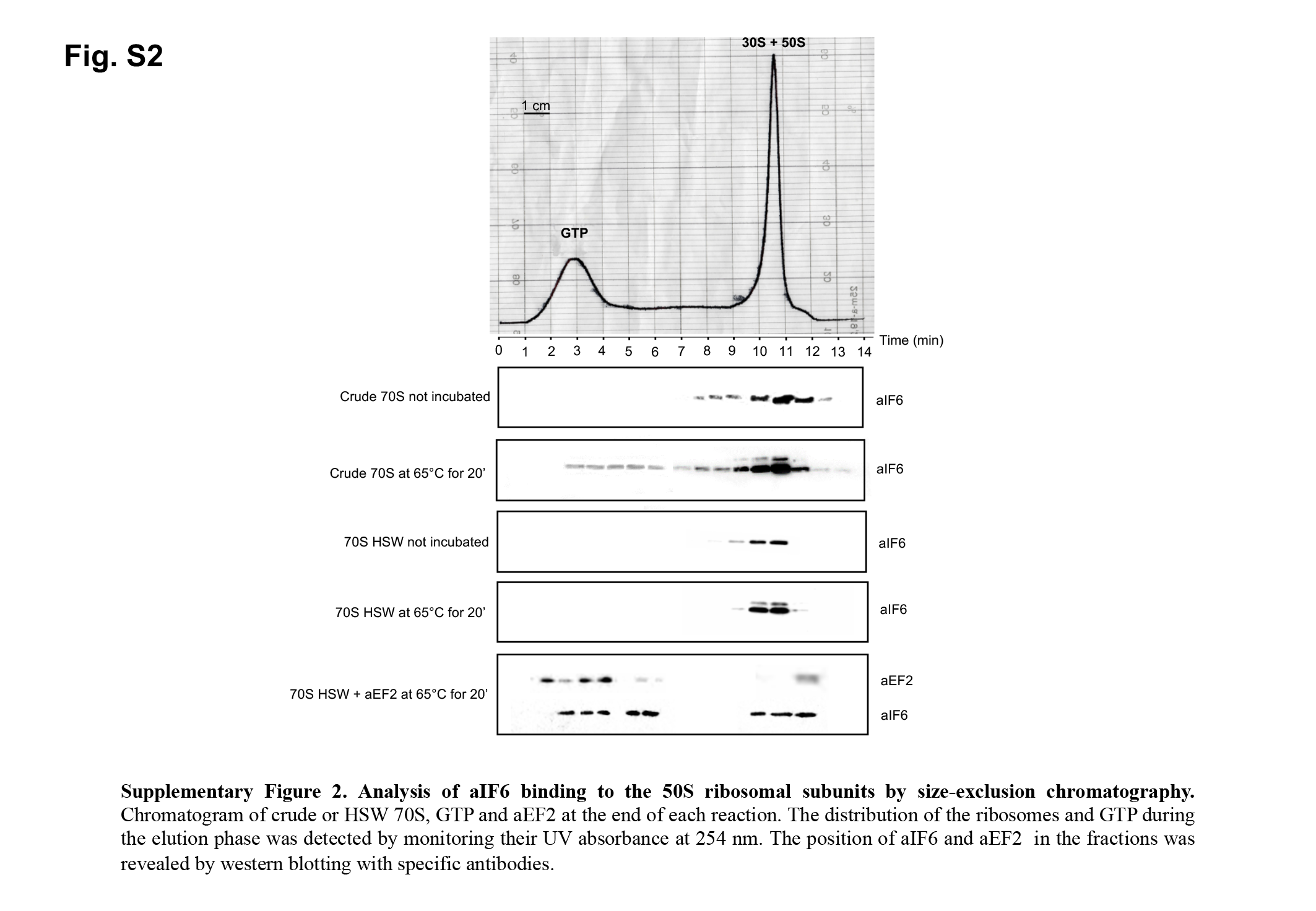

Supplement: Supplementary file 2 [file Image_2.TIF]

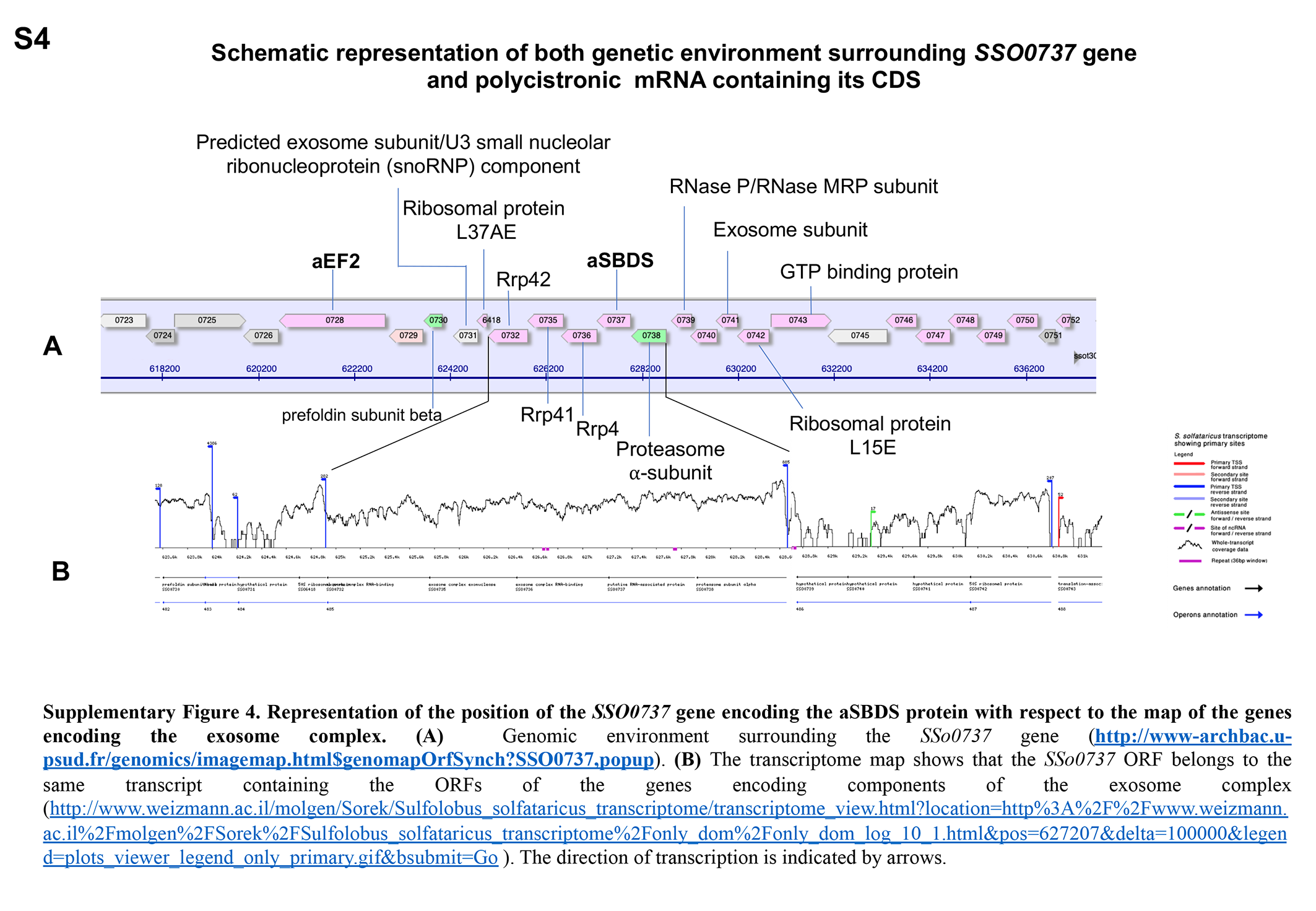

Supplement: Supplementary file 4 [file Image_4.TIF]
