# Supplementary material for: The Archaeal Elongation Factor EF-2 Induces the Release of aIF6 From 50S Ribosomal Subunit
Source: Front Microbiol. 2021 Mar 24;12:631297. doi: 10.3389/fmicb.2021.631297 (PMC8024482; doi:10.3389/fmicb.2021.631297)
Supplement: Supplementary file 3 [file Image_3.pdf]

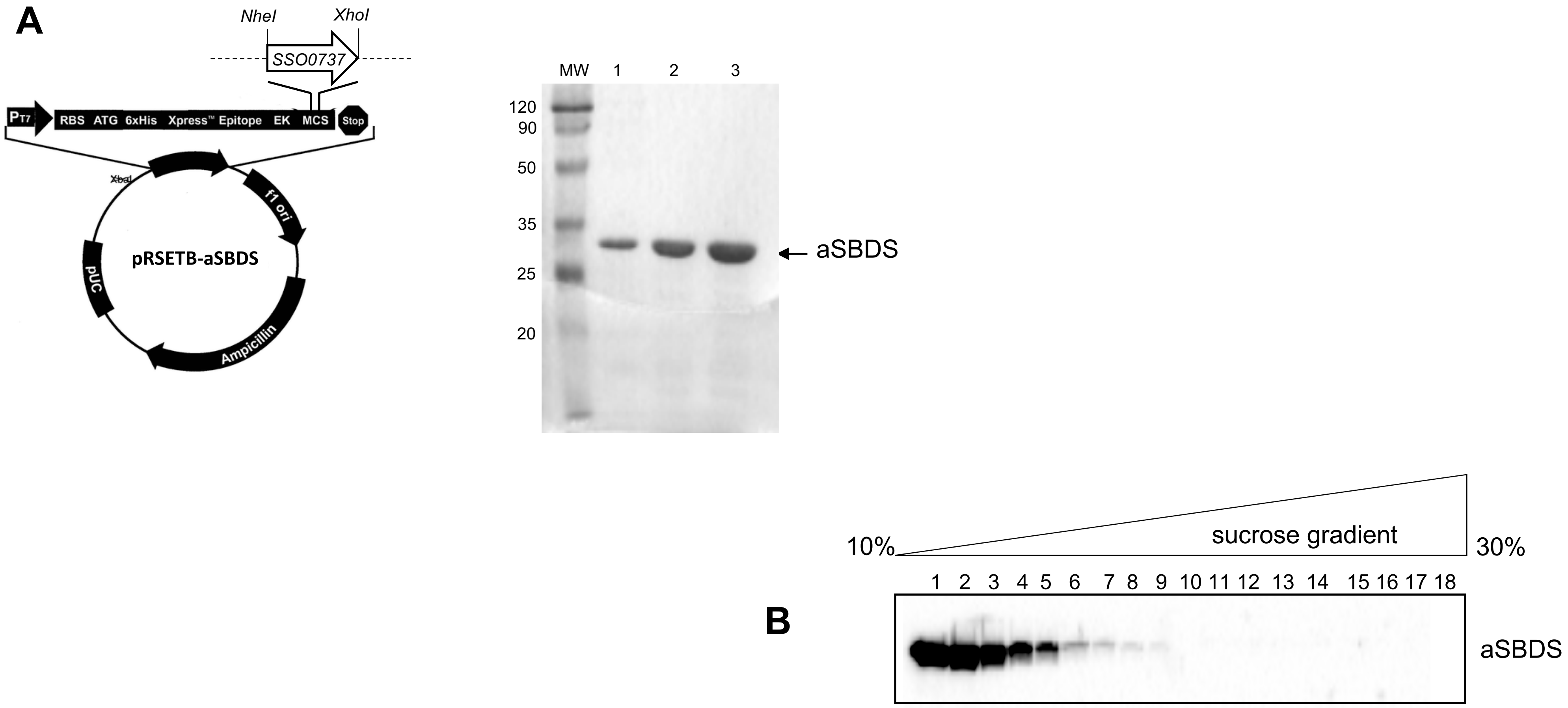


**Supplementary Figure 3. *In vitro* expression of *Sulfolobus solfataricus* gene *SSO0737* encoding the aSBDS protein and its sedimentation along a 10-30% sucrose gradient.** (A) On the left, a schematic representation of the pRSETB-aSBDS construct. It was designated by amplifying the gene of our interest with the primers described in Materials and Methods section. The PCR product, a DNA fragment of 732 bp, was introduced into the *NheI*-*XhoI* restriction sites of the pRSETB expression plasmid. The coding region starts with the ATG codon provided by the plasmid and preceding a region coding for six histidines (6His-tag). On the right, coomassie-stained SDS-PAGE showing 1, 2 and 4 µl of eluted protein (lane 1, 2 and 3, respectively) recovered after heat-treatment and affinity chromatography on Ni-NTA agarose of *E.coli* BL21(DE3) cell lysates containing the pRSETB-aSBDS plasmid. (B) Density gradient fractionation of recombinant aSBDS (100 pmol) incubated with GTP at 65°C for 20 min. The position of the protein was revealed by western blotting of the individual fractions with the anti-aSBDS antibody.
